# Case Report: Stage IIIc primary malignant mixed Müllerian tumor of the fallopian tube: A case of 5-year disease-free survival after cytoreductive surgery combined with peritoneal resection and adjuvant chemotherapy with paclitaxel plus carboplatin

**DOI:** 10.3389/fonc.2022.1054307

**Published:** 2022-12-22

**Authors:** Xiaoli Xiao, Ruiqing Ma, Lianyao Shi, Cong Wang, Jie Chen, Yiyan Lu, Yufeng Wang, Xichao Zhai, Fengxian Fu

**Affiliations:** ^1^ Department of Gynecology, Aerospace Center Hospital, Beijing, China; ^2^ Department of Myxoma, Aerospace Center Hospital, Beijing, China; ^3^ Department of Imaging, Aerospace Center Hospital, Beijing, China; ^4^ Department of Pathology, Aerospace Center Hospital, Beijing, China; ^5^ Department of Ultrasonography, Aerospace Center Hospital, Beijing, China

**Keywords:** malignant mixed Müllerian tumor (MMMT), cytoreductive surgery (CRS), cytoreduction score, tumor-free survival, postoperative chemotherapy (POCT)

## Abstract

Malignant mixed Müllerian tumor (MMMT) of the fallopian tube is rare and has a poor prognosis. For the patient with fallopian tube MMMT, complete resection of the tumor invading the viscera and the peritoneum is a prerequisite for long-term survival. We report a case of stage IIIc MMMT of the fallopian tube treated by cytoreductive surgery (CRS), peritoneal resection, and adjuvant chemotherapy (paclitaxel plus carboplatin), with 5-year tumor-free survival. Postoperative chemotherapy combining platinum and paclitaxel is the most potent adjuvant therapy.

## Introduction

MMMT accounts for 0.1% to 0.5% of all gynecologic malignancies (1). It is common in the uterus, cervix, and ovary but extremely rare in the fallopian tube. By early 2022, 95 cases of malignant mixed Müllerian tumors of the fallopian tube had been reported in the literature (2), but no standardized treatment approach exists. In this paper, we report for the first time a case of stage III primary MMMT of the fallopian tube who underwent cytoreductive surgery (CRS), peritoneal resection, and adjuvant chemotherapy with paclitaxel plus carboplatin five years earlier. A prerequisite for long-term survival is complete macroscopic resection of the malignancy, requiring the removal of the tumor invading the viscera and the area of the peritoneum infiltrated by the cancer. Our experience in treating this case can provide valuable information about MMMT of the fallopian tube.

## Case report

Primary MMMT of the fallopian tube mainly occurs in the fifth or sixth decade of life, the average age of postmenopausal women (3). A 54-year-old Chinese female presented to the hospital in October 2017, complaining of progressive lower abdominal pain for one year and an enlarged abdomen for two months. Her medical history showed bilateral tubal sterilization in 1988 and the last menstruation on May 1, 2017. She had also experienced apopsychia and a 5-kg weight loss. She had received anti-inflammatory therapy, hysteroscopic endometrial polypectomy, and GnRH-a twice for a misrecognized hysteromyoma. However, her symptoms did not improve significantly.

**Figure 1:**
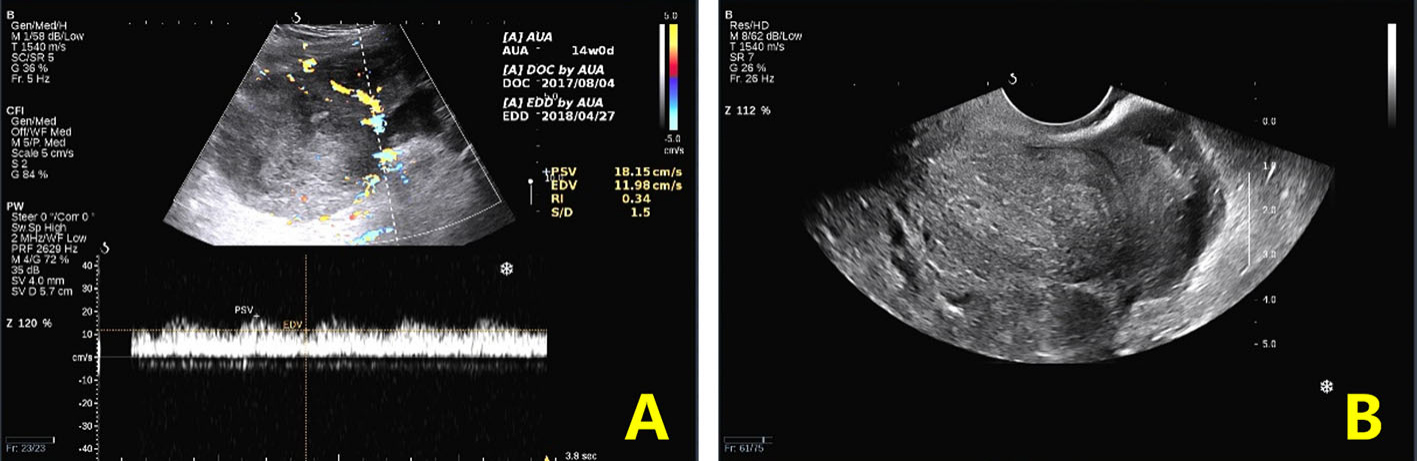
Preoperative ultrasound findings. **(A)**. Transabdominal ultrasound: cross-section view shows a heterogeneous echogenic mass and internal venous and low-resistance arterial blood flow spectrum. **(B)**. Transvaginal ultrasound: longitudinal section of the uterus showing a posterior uterus in the center surrounded by heterogeneous echoes.

**Figure 2:**
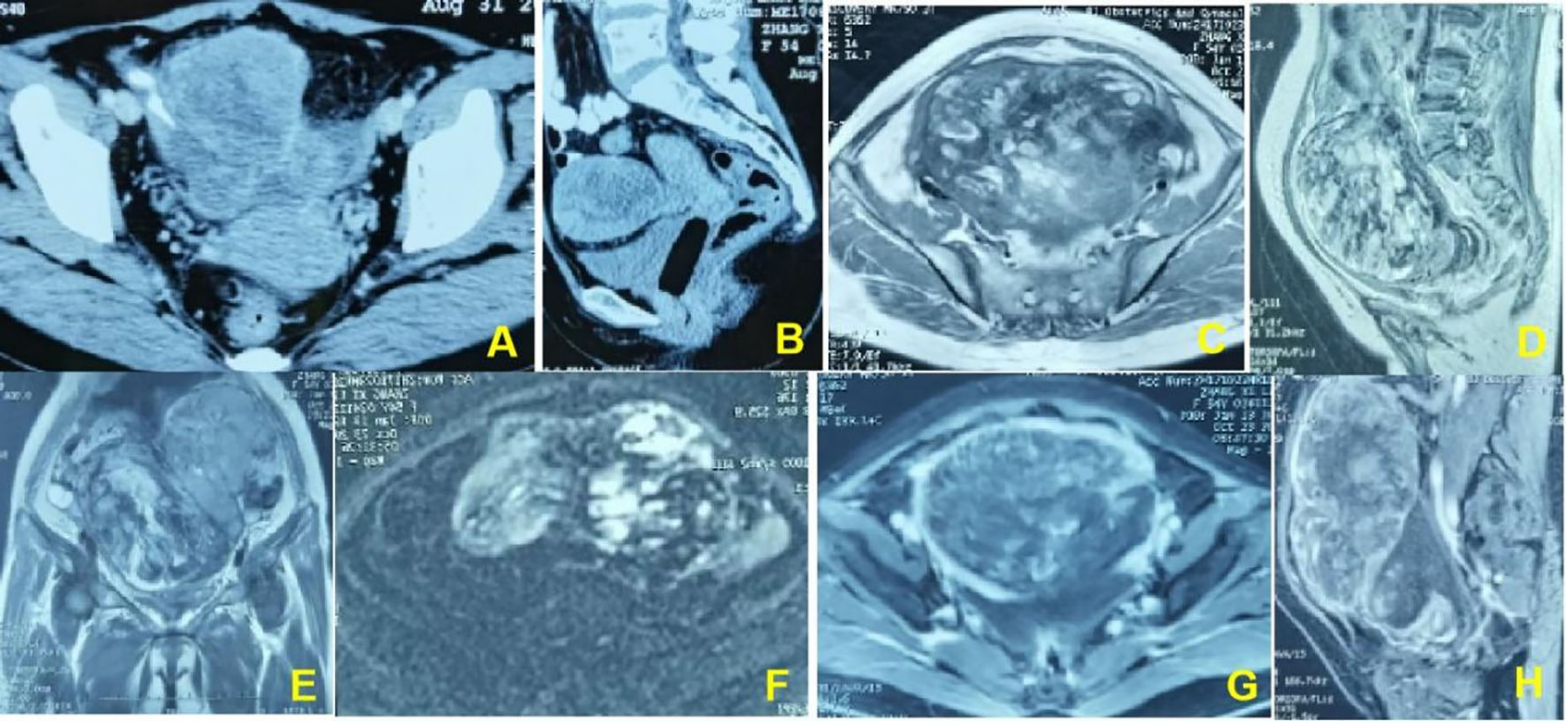
**(A, B)**. In a 54-year-old woman, pelvic CT enhancement suggests clumpy soft-tissue foci with uneven internal enhancement and a central necrotic zone. **(C–E)**. Pelvic magnetic resonance T2WI shows an uneven internal signal of the lesion and multiple small cysts inside the lesion. **(F)**. The DWI sequence shows multiple flaky hyperinflections inside the lesion. **(G, H)**. The enhancement sequence shows significantly uneven enhancement in the lesion and more pronounced peripheral intensification.

Physical examination revealed a large mass measuring 15*15 cm in the abdomen, with tenderness and rebound in the suprapubic area. Ultrasound, pelvic magnetic resonance imaging (MRI), and computed tomography (CT) revealed seroperitoneum and a complex mass measuring 15*14.7*11 cm anterior to the uterine fundus, suggestive of hemoperitoneum (probably originating from the rupture of a uterine mass) and sarcoma (**Figures 1**, **2**). The cancer antigen 125 (CA 125) level was 424.10 U/mL. The hemoglobin level was 6.0 g/dL, requiring six units of red blood cell suspension to correct her anemia. On October 31, 2017, the patient underwent exploratory laparotomy, and approximately 100 ml of hemoperitoneum was identified. A large cystic, solid tumor measuring 20*20*15 cm was seen in the pelvis, with a structurally normal uterus but missing bilateral appendages. The tumor was extensively and densely adherent to the pelvic peritoneum, omentum, small intestine, and part of the sigmoid colon. The rectum and bladder were also extensively and densely adherent, and the greater omentum was pie-shaped. A 2 cm incision was made in the tumor cyst wall, and about 1000 ml of viscous pus and blood-like fluid was aspirated. After the tumor cyst wall incision was enlarged, a large amount of yellow-green pus and a moss-like substance was found inside the tumor, along with papillary hyperplasia of the cyst wall and extensive rotten fish-like brittle tissue in the cyst.

**Figure 3:**
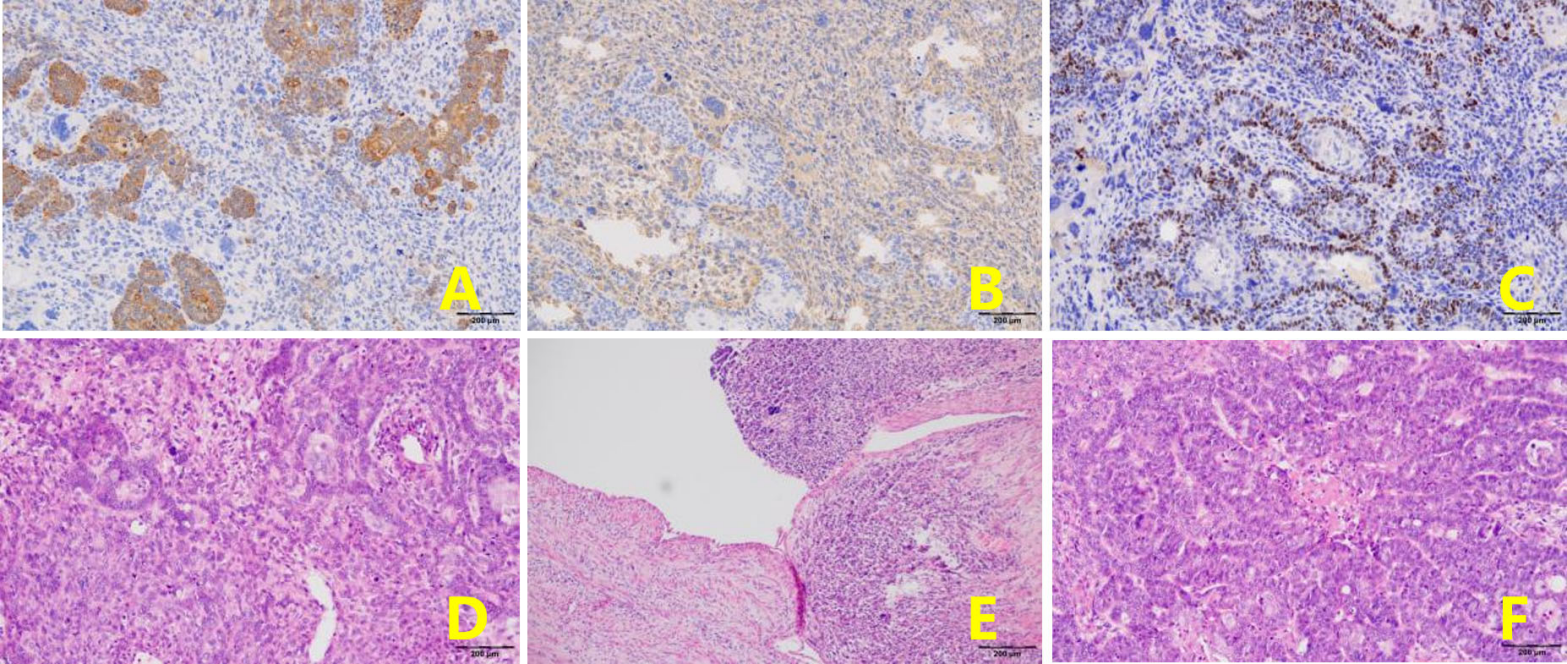
The same type of infiltrative tumor was found in both fallopian tubes, containing poorly differentiated adenosquamous cell carcinoma and undifferentiated sarcoma. **(A)**. CK + (epithelial component) (EnVision, x100). **(B)**. Vimentin+ (mesenchymal component) (EnVision, x100). **(C)**. P40+ (squamous carcinoma component) (EnVision, x100). **(D)**. The tumor consists of epithelial and mesenchymal components (H&E, ×100). The mesenchymal component is an undifferentiated sarcoma with marked nuclear atypia (H&E, ×100). **(E)**. Tumor tissue has migrated with bilateral tubal mucosa (H&E, ×100). **(F)**. Adenocarcinoma carcinoma with marked nuclear atypia (H&E, ×100).

**Figure 4:**
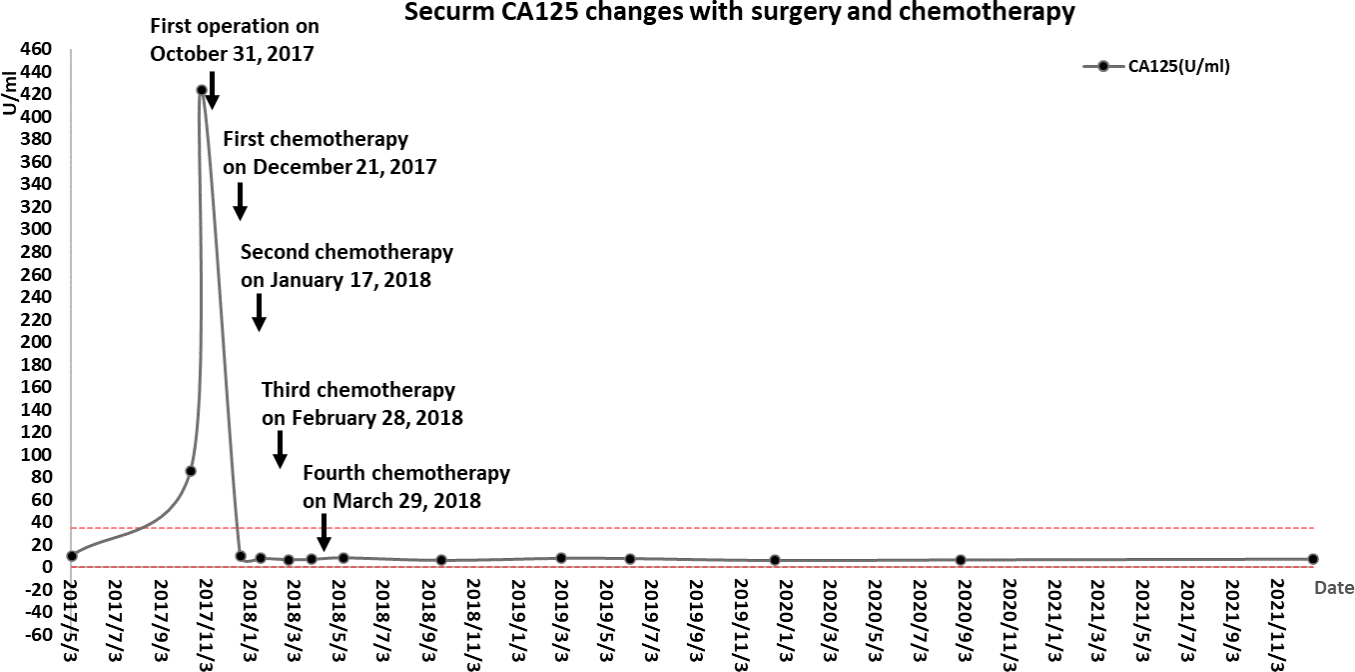
Changes in serum tumor markers.

Part of the collected tissue samples, prepared into frozen sections, confirmed malignancy. This patient had extensive dense adhesions between the pelvic tumor and pelvic peritoneum, which were difficult to separate. Meanwhile, the pelvic anatomy was destroyed due to the invasion of the tumor, so it was impossible to determine the presence or absence of pelvic peritoneal metastasis. A CRS was planned to remove as much tumor tissue as possible and prevent peritoneal metastasis caused by surgery. A series of procedures were performed, including extraperitoneal total hysterectomy, bilateral oophorectomy, pelvic peritonectomy with preservation of the rectum and sigmoid colon, high ligation of the infundibulopelvic ligament, omentectomy, appendectomy, and bowel surface lesion resection. The lateral pelvic peritoneum was separated from the abdominal wall, the round uterine ligament and uterine arteries were isolated from the outer peritoneum, the ureteral pelvic segment was exposed and protected, and separation continued along the pelvic floor peritoneum to the margin of the upper rectum. Finally, the vagina was cut off along the vaginal dome, removing the peritoneum on the posterior vaginal wall, rectal surface, and the Pouch of Douglas. A layer of adipose tissue covered the serosa of the mid-rectum, protecting the intestine from damage by peritonectomy. In contrast, the serous membrane of the upper rectum and sigmoid colon is the visceral peritoneum; therefore, peritonectomy was not performed in this case, thereby avoiding postoperative complications. No intraoperative complications and residual tumors in the abdomen were observed. Microscopically, the same type of infiltrative tumor was found in both fallopian tubes, containing poorly differentiated adenosquamous cell carcinoma (accounting for 80%) and undifferentiated sarcoma involving the surface of the right ovary, pelvic peritoneum, and greater omentum. The uterus, surface lesions of the intestinal tract, and appendix were tumor-free. Immunohistochemistry showed tumor cells: HER-1 (2+), HER-2 (-), Ki-67 (+ >75%), P53 (+ >75%), Top IIα (+ 50-75%), CK (cancer component +), Vimentin (sarcoma component +), ER (2+ <5%), PR (-), WT-1 (-), PAX-8 (-), NapsinA (-), CK5/6 (Squamous cell element+), P40 (squamous cell element+), P63 (squamous cell element+), CDX2(-), AFP(-), CD99(+), HCG-β(-), CD30(-), EMA (cancerous component foci+), SMA(-), Desmin(-), MyoD1(-), S-100(-), HMB45(-), MelanA(-), CD68 (sarcomatous component foci+). The tumor had migrated with the bilateral fallopian tube mucosa, indicating its origin in the fallopian tube (**Figure 3**). Postoperative diagnosis: stage IIIc MMMT of the fallopian tube. From December 21, 2017, to March 29, 2018, the patient was treated with four cycles of intravenous chemotherapy based on carboplatin (500 mg) and paclitaxel (210 mg), with an interval of about one month. During chemotherapy, the CA125 returned to normal (**Figure 4**), but the patient refused to continue chemotherapy because of bone marrow IV suppression. So far, no recurrence has been detected during the follow-up period.

## Discussion

MMMT contains both stromal and epithelial elements and carcinomatous and sarcomatous elements. It typically has a high grade, an aggressive progression, and a poor prognosis. According to the components in the tumor, carcinoma is classified into adenocarcinoma, clear cell carcinoma, serous papillary carcinoma, or rare squamous cell carcinoma. Sarcoma contains homologous components, such as muscle or mesenchyme, or heterologous components, such as bone, cartilage, or non-smooth muscle fibers. In our case, the tumor was composed of poorly differentiated adenosquamous cell carcinoma (80%) and undifferentiated sarcoma, which is extremely rare and highly malignant.

The clinical symptoms of sarcoma of the fallopian tube include abdominal pain in the hypogastric area (47.9%), abnormal vaginal bleeding (34.2%), and/or pelvic mass (6.9%) (1). In its advanced stage, urinary, gastrointestinal, or respiratory symptoms may appear due to non-specific metastatic lesions. No specific tumor markers can successfully predict MMMT. The serum CA-125 may rise, but its relationship with MMMT status has no evidence base. It is difficult to differentiate MMMT of the fallopian tube from hydrosalpinx, ovarian malignancy, or tuboovarian abscess before surgery. Transvaginal ultrasonography, CT scanning, or MRI can help render a diagnosis, but all lack specificity and accuracy. Due to these reasons, diagnosing MMMT is challenging and always established by postoperative histology. In the present case, only abdominal pain occurred as the primary symptom. Its aggravation indicated gradual enlargement of the pelvic mass and progressive increase of serum CA-125. It may be inferred that a high serum CA-125 reflects rapid disease progression. Atypical clinical symptoms and imaging characteristics may lead to the misdiagnosis of MMMT as uterine leiomyoma, which led to 2 cycles of GnRH-a administration.

A Medline search (2) revealed 94 patients reported between 1902 and 2019, plus 1 case reported in the article: a total of 95 cases. Ten cases were excluded because the patients were lost to follow-up or lacked relative information. FIGO stage I was identified in 17 cases, stage II in13 cases, stage III in 43, and stage IV in 10 cases and not determined in 2 cases. The 85 cases were divided into two groups: The no evidence of disease group, including 34 patients without any residual disease at the end of the follow-up period; the non-survival group, including 51 patients who died from fallopian tube carcinosarcoma or its complications(**Table 1**). For patients aged between 41 and 60 years,the symptoms at presentation and CT/MRI tumor evidence were considered prognostic factors. Omentectomy and pelvic lymphadenectomy were found to be significant factors for survival. Fimbrial localization and the heterologous type of tumor were negative prognostic factors. Chemotherapy was found to improve survival (**Table 2**).

**Table 1:** Previously reported cases of fallopian MMMT (2).

Patient No.	Author (Refs.)	Year of report	Age of patients (years)	FIGO stage	Outcome
1	Motta	1926	14	IV	DOD
2	Zacho	1933	N/D	IIIC	DOD
3	Platz	1940	58	IV	DOD
4	Bochner	1961	58	N/D	DOD
5	Williams and Woodruff	1963	35	IV	DOD
6	Malnasy and Gaal	1963	45	IIB	DOD
7	McQueeney et al	1964	69	IIB	DOD
8	De Queiroz and Roth	1970	64	IIIC	DOD
9	Wu et al	1973	57	IA	NED
10	Acosta et al	1974	46	IIB	DOD
11	Acosta et al	1974	62	IV	DOD
12	Acosta et al	1974	48	IC	DOD
13	Aggarwal et al	1976	50	IIIC	DOD
14	Manes and Taylor	1976	76	IA	DOD
15	Manes and Taylor	1976	74	IA	DOD
16	Manes and Taylor	1976	47	IA	NED
17	Manes and Taylor	1976	58	IA	NED
18	Henderson et al	1977	62	IIB	DOD
19	Jain	1977	52	IA	NED
20	Oka et al	1978	57	IA	NED
21	Hanjani et al	1980	62	IV	DOD
22	Viniker et al	1980	63	IA	NED
23	Holst and Erichsen	1981	65	IIIC	NED
24	O’Toole et al	1982	71	IV	DOD
25	Egorov	1982	53	N/D	DOD
26	Kahanpää et al	1983	65	III	NED
27	Deppe et al	1984	68	IIIB	NED
28	Punnonen et al	1985	68	IIIB	DOD
29	Buchino and Buchino	1987	61	IIIC	DOD
30	Yabushita et al	1987	53	IIA	NED
31	Chen and Wolk	1988	56	IC	DOD
32	Muntz et al	1989	57	IIIC	DOD
33	Muntz et al	1989	60	IIIA	DOD
34	Muntz et al	1989	61	IV	DOD
35	Axelrod et al	1989	62	IIIC	NED
36	Kinoshita et al	1989	79	IC	NED
37	van Dijk et al	1990	45	IIA	DOD
38	van Dijk et al	1990	67	IIIB	DOD
39	Seraj et al	1990	62	IIIC	DOD
40	Seraj et al	1990	53	IIIC	DOD
41	Liang et al	1990	63	IIIC	DOD
42	Chang et al	1991	66	III	DOD
43	Chiou et al	1991	63	IIIC	DOD
44	Imachi et al	1942	60	IIIC	DOD
45	Imachi et al	1942	67	IV	DOD
46	Moore et al	1942	66	IIIC	DOD
47	Carlson et al	1993	72	IIIC	DOD
48	Carlson et al	1993	56	IIIC	NED
49	Carlson et al	1993	60	IB	NED
50	Carlson et al	1993	44	IA	NED
51	Carlson et al	1993	59	IIIB	NED
52	Weber et al	1993	74	IIA	NED
53	Zorlu et al	1994	38	III	DOD
54	Horn et al	1996	62	IIIB	DOD
55	Horn et al	1996	64	IIB	DOD
56	Horn et al	1996	69	IIIC	DOD
57	Horn et al	1996	71	IV	DOD
58	Ebert et al	1998	70	IA	NED
59	Maitra et al	2004	29	IIIA	DOD
60	Moustafa et al	2004	75	IIA	DOD
61	Humble and Carter	2004	63	IIIC	DOD
62	Lim et al	2004	57	IA	NED
63	Gagner and Mittal	2005	77	IV	DOD
64	Kuroda et al	2005	65	IIIB	DOD
65	Das et al	2005	49	III	NED
66	Das et al	2005	80	IIB	DOD
67	Hudelist et al	2006	57	IIB	NED
68	Kuroda et al	2007	77	IIIC	DOD
69	Kawaguchi et al	2008	69	IC	NED
70	Kourea et al	2008	72	IIIC	NED
71	Piura et al	2009	46	IIIC	NED
72	Shen et al	2010	55	IIIC	NED
73	Shen et al	2010	46	IIIC	DOD
74	Malhotra et al	2012	58	IIIC	DOD
75	Watanabe et al	2012	60	IIIC	NED
76	Tsai et al	2012	53	IIIA	NED
77	Gupta and Jenison	2011	57	IIIC	DOD
78	Takemoto et al	2015	74	IIIC	DOD
79	Narin et al	2015	56	IIA	NED
80	Vale-Fernandes et al	2015	68	IIA	NED
81	Ji et al	2015	57	IIIC	NED
82	Monsalve et al	2015	71	III	NED
83	Zhang et al	2018	70	IIIB	NED
84	Bécsi et al	2019	70	IIIB	NED
85	Cozlea et al	2022	65	IC	NED

NED, no evidence of disease; DOD, death of disease; N/D, not determined.

**Table 2:** The features of 85 cases of fallopian MMMT and follow-up.

Features	NED group n=34, n (%)	DOD group n=51, n (%)
Age of the patients (years)		
<40	0 (0)	4 (5.9)
41-60	19 (55.9)	16 (31.4)*
61-80	15 (44.1)	31 (60.8)
Abdominal distention	5(14.7)	18 (35.3) *
CT/MRI tumor evidence	11 (32.4)	5 (9.8) *
Surgical procedure		
Omentectomy	21 (61.8)	18 (35.3) *
Pelvic lymphadenectomy	15 (44.1)	11 (21.6)
Presence of extragenital metastases		
Lymph nodes	5 (15.2)	18 (35.3) *
Bowel	2 (6.1)	11 (21.6)
Distant	0 (0)	9 (17.6) *
FIGO staging		
I (A-C)	13 (38.2)	4 (7.8) *
II (A-B)	5(14.7)	8 (15.7)
III (A-C)	16 (47.1)	27 (52.9)
IV	0 (0)	10 (19.6) *
Histological type		
Homologous	17 (51.5)	15 (29.4) *
Heterologous	15 (45.5)	36 (70.6) *
Liposarcoma, angiosarcoma	4 (12.1)	0 (0) *
Chemotherapy		
Received	24 (72.7)	20 (39.2) *
Not received	9 (27.3)	28 (54.9) *
First-line chemotherapy agents		
Carboplatin+paclitaxel	11 (45.8)	6 (30.0) *

*P < 0.05.

The survival rate was 0.9879 at one month, 0.8049 at six months, 0.4657 at two years, and 0.2865 at five years of follow-up. A standard therapeutic approach has not been established for fallopian tube carcinosarcoma (2). Hysterectomy and bilateral salpingo-oophorectomy are performed for most of these cases. Omentectomy and pelvic lymphadenectomy can prolong survival, but other surgical procedures, such as appendectomy, paraaortic lymphadenectomy, peritonectomy, or metastasis resection, do not significantly affect the outcome. Reviewing these cases, we failed to confirm the relationship between the prognosis and the metastasis to the greater omentum, peritoneum, and paraaortic lymph nodes. Therefore, it cannot be proven that resection of these sites benefits cytoreductive surgery. However, peritoneal metastasis is the inevitable pathological process for advanced ovarian cancer, and most patients with serous ovarian carcinoma have progressed to clinical stage III by the time they seek medical treatment. Ovarian cancer tends to metastasize along the peritoneal surface involving the pelvic and abdominal peritoneum, including the omentum, peritoneal surface of the small intestine and colon, mesenterium, peritoneum of colon gutters, diaphragm, and surfaces of the liver and spleen in two-thirds of the patients with ascites. These patients make up a suitable population for CRS (4). The treatment strategy for fallopian tube MMMT is similar to that for ovarian cancer. Surgical resection and platinum-based chemotherapy have been successfully used in many patients. Undoubtedly, resecting all the lesions to the greatest extent is a critical step to good clinical outcomes. Lately, many studies have emerged supporting this point of view (5, 6). Thus, the current standard therapy for primary fallopian MMMTs is staging laparotomy or cytoreductive surgery combined with postoperative chemotherapy based on paclitaxel and carboplatin. The same regimen can be used for ovarian epithelial carcinoma. Staging laparotomy should include the collection of flush fluid and/or ascites, thorough exploration of all peritoneal surfaces, omentectomy, appendectomy, pelvic lymph node sampling, and peritoneal biopsies (as necessary).

Aggressive CRS, aiming to remove as much tumor tissue as possible, is warranted in patients with advanced disease. The cytoreduction (CC) score completeness is a major prognostic factor for peritoneal cancer patients, and it is suitable for ovarian cancer with peritoneal metastasis (7). The CC has become an objective quantitative index for evaluating the effect of tumor resection: CC-0, no residual tumor nodule after cytoreduction; CC-1: residual tumor diameter<2.5 mm; CC-2: residual tumor diameter 2.5 mm-2.5 cm; and CC-3: residual tumor diameter >2.5 cm or the residual tumor cannot be removed or palliatively removed. Besides, the CC is also a part of CRS. The standardized CRS and complete resection of all visible malignant tumors are the basis of long-term survival (8). Peritoneal metastases and severe adhesions, even structural disorders, are the obstacles to achieving CC-0. Sugarbaker elaborated standardized CRS during PMP peritonectomy as early as 1995 (9). With peritonectomy as the core technique, the complete resection of macroscopic malignancy, which requires removal of the tumor invading the viscera and the peritoneum infiltrated by the tumor, serves as the basis for long-term survival.

To remove as much tumor tissue as possible, the patient underwent extraperitoneal CRS and achieved CC-0. The peritoneal cancer index (PCI) score can evaluate tumor burden during comprehensive abdominal exploration and define which regions in the peritoneum should be removed or stripped and whether an optimal CRS can be performed (10). The PCI in the current case was 9. Optimal CRS helped to achieve CC-0, which may be attributed to the technical specifications for peritoneal resection in the pelvic peritoneum. To remove tumors implanted on the peritoneum, complete CRS may require an extraperitoneal approach, which can engender a higher resection rate.

Systemic chemotherapy significantly improves survival, mainly associated with an optimal CRS (5). Multiple regimens have demonstrated platinum-based chemotherapy as the most potent therapy for fallopian MMMTs (5). Because the peritoneum and greater omentum were positive for carcinosarcoma in our patient, stage IIIc fallopian tube MMMT was diagnosed. Fallopian tube MMMT containing poorly differentiated carcinoma and undifferentiated sarcoma is highly aggressive, with poor survival of 16.1 months on average (6).

**Figure 5:**
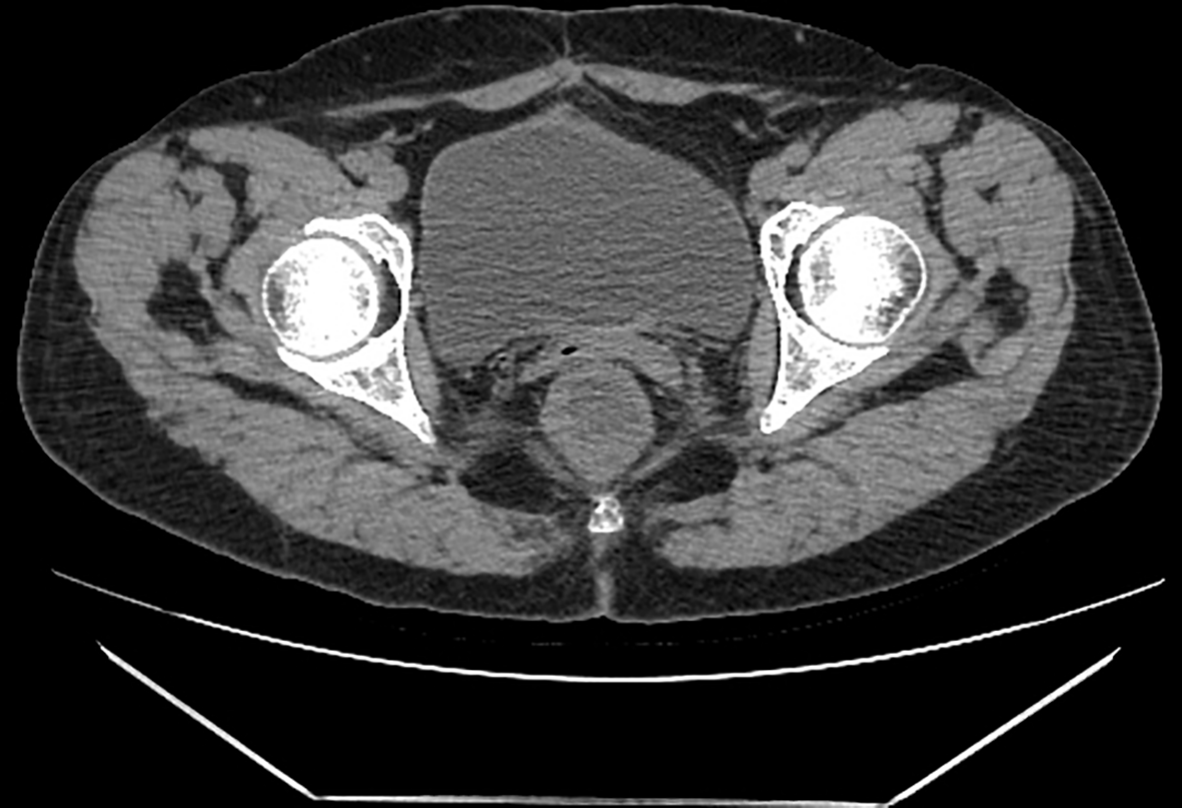
Postoperative CT findings. The patient was reexamined by pelvic CT five years after surgery, and no recurrence or metastasiswas found in the pelvic cavity.

Four courses of paclitaxel and carboplatin therapy were given monthly instead of every three weeks after peritonectomy combined with CRS. The patient terminated the remaining two courses because of severe myelosuppression. At the time of information, the patient had lived disease-free for five years after therapy (**Figures 4**, **5**). The combination of paclitaxel and carboplatin has been widely used in various malignant gynecological diseases due to its high activity and acceptable toxicity. Three meta-analyses have also revealed that this combination is associated with more prolonged survival (2, 5, 11).

## Conclusion

Fallopian MMMTs should be a differential diagnosis in all postmenopausal patients who present with pelvic masses, vaginal bleeding, abdominal pain, or distension but no other significant findings. Due to its non-specific presentations, symptomatology, and low incidence, large-size randomized trials should be conducted to explore effective treatment options and establish a consensus. For the patient with fallopian tube MMMT, complete resection of the macroscopic malignancy, which requires the removal of the tumor invading the viscera and the peritoneum infiltrated by the tumor, is a prerequisite for long-term survival. Postoperative chemotherapy combining platinum and paclitaxel is the most potent adjuvant therapy. This case report provides new information regarding the diagnosis, treatment, and prognosis of MMMTs. Others may replicate our experience to improve survival and quality of life in patients with MMMTs.

## Data availability statement

The raw data supporting the conclusions of this article will be made available by the authors, without undue reservation.

## Ethics statement

The studies involving human participants were reviewed and approved by Aerospace Center Hospital Ethics committee. The patients/participants provided their written informed consent to participate in this study.

## Author contributions

XX and LS provided study material or patients; CW, YW, YL, and JC collected and analyzed the data; XX and RM contributed to the draft of the manuscript; FF and XZ revised the manuscript critically for important intellectual content. All authors approved the final version of the manuscript to be submitted. All authors agreed to be accountable for all aspects of the work; ensuring that questions related to the accuracy or integrity of any part of the work are appropriately investigated and resolved.
